# The Black Drop, an Old Preparation

**Published:** 1826-07

**Authors:** 


18. *Black Drop.* Any man of leisure, and of even very moderate research, might furnish the public with a series of papers, (having for their motto, "*suum cuique*," and their *object*, that which is conveyed in those two significant words) which would be of real utility—and moreover extremely amusing. It is not in our power to undertake this measure, in its full extent—but we invite communications on this subject—and, in the next Number of this Journal, a series of *retrospections* will be commenced, which will probably be of some interest.

At present we shall only advert to a medicine which is unquestionably of very great importance, and the real composition of which is yet a secret. Public attention was drawn to the Black, or Lancaster Drop, about ten years ago, by Dr. Armstrong, who published the formula in the first edition of his valuable work on typhus fever. It is certain, however, that full 21 years ago, two formulæ were given to the public by a Dr. James Cassels of Lancaster—one corresponding with that of Dr. Armstrong, and the other as follows:—"take of purified opium five ounces—pimento and cinnamon of each two drachms—saffron and seville orange peel, of each one drachm—rectified spirit of wine a pint. Digest with a gentle heat for a week, and strain the liquor through flannel with a screw-press; then add two or three ounces of sugar-candy."

A friend of ours made the Black Drop according to the other prescription (with the verjuice) but it did not correspond in colour, taste, or property, with that which is sold as a secret preparation. We would therefore recommend a trial of the last-mentioned formula, as a succedaneum for a very expensive, but very valuable medicine.

It is a fact that neither the one nor the other formula is original; for Dr. Jones, in his "Mysteries of Opium revealed," published nearly one hundred and thirty years ago, makes mention of a preparation of opium similar to Dr. Armstrong's receipt, under the title of "*Laudanum Liquidum Cydoneatum.*" Quincy, in his Dispensatory, published in 1722, takes notice of this formula, and gives others for making laudanum with spices and aromatics, similar to the form which we now republish.

